# Effect of the circadian cycle in Late Asthmatic Response (LAR). Comparison between two allergic asthma models in rats and mice

**DOI:** 10.1186/1476-9255-10-S1-P13

**Published:** 2013-08-14

**Authors:** Raquel Otal, Sandra Muñoz, Elena Calama, Isabel Pagán, Félix Gil, Núria Torán, Montserrat Miralpeix, Jorge De Alba

**Affiliations:** 1 Respiratory Therapeutic Area, Discovery, Almirall R&D Centre, 08980 Barcelona, Spain

## 

The ovalbumin (OVA) model of allergic inflammation, both in rat and mouse is characterised by a late bronchoconstrictive response (LAR) after allergen challenge that can be measured using whole-body plethysmography (WBP) in conscious unrestrained animals.

Circadian oscillations of lung mechanical properties have been reported in conscious undisturbed rodents. Potentially, these fluctuations could alter the pulmonary response induced by allergen provocation and the response of pharmacological treatments.

This circadian influence has been showed in asthma patients [1], where an allergen inhalation challenge given in the evening produced a more frequent and severe LAR compared to that one given in the morning.

The aim of this study was to assess whether LAR in OVA-sensitized rats and mice was affected differently when animals were exposed to allergen in different times of day.

Firstly, to confirm the circadian oscillations, changes in several respiratory parameters were monitored during 24h in naïve Brown Norway rats or C57/Black6 mice. Afterwards, OVA-sensitized animals were exposed to the allergen in different periods of the day and the pulmonary function parameters were monitored, including the LAR.

In rats, LAR evoked in three different times of day (morning, afternoon or evening) showed no differences in severity, but was more frequent during the evening. In contrast, the LAR triggered in mice in the morning was more severe and more frequent than that evoked during the evening.

These results suggest that species and day period of time to induce LAR in allergic animal models are critical and should be taken into account when evaluating the effects of new compounds on this read-out.

## References

[B1] Mohiuddin AA Martin RJ Circadian basis of the late asthmatic response Am Rev Respir Dis 1990 142 5 1153 7 10.1164/ajrccm/142.5.1153 2240837

